# Enhancing Superconductivity of the Nonmagnetic Quasiskutterudites by Atomic Disorder

**DOI:** 10.3390/ma13245830

**Published:** 2020-12-21

**Authors:** Andrzej Ślebarski, Maciej M. Maśka

**Affiliations:** 1 Institute of Physics, University of Silesia in Katowice, 75 Pułku Piechoty 1, 41-500 Chorzów, Poland; 2 Centre for Advanced Materials and Smart Structures, Polish Academy of Sciences, Okólna 2, 50-950 Wrocław, Poland; 3 Department of Theoretical Physics, Wroclaw University of Science and Technology, Wybrzeże Wyspiańskiego 27, 50-370 Wrocław, Poland; maciej.maska@pwr.edu.pl

**Keywords:** skutterudite-related superconductors, superconductivity, BCS theory, atomic disorder

## Abstract

We investigated the effect of enhancement of superconducting transition temperature $T_{c}$ by nonmagnetic atom disorder in the series of filled skutterudite-related compounds (La${}_{3}M_{4}$Sn${}_{13}$, Ca${}_{3}$Rh${}_{4}$Sn${}_{13}$, Y${}_{5}$Rh${}_{6}$Sn${}_{18}$, Lu${}_{5}$Rh${}_{6}$Sn${}_{18}$; M= Co, Ru, Rh), where the atomic disorder is generated by various defects or doping. We have shown that the disorder on the coherence length scale ξ in these nonmagnetic quasiskutterudite superconductors additionally generates a non-homogeneous, *high-temperature* superconducting phase with $T_{c}^{⋆}>T_{c}$ (dilute disorder scenario), while the strong fluctuations of stoichiometry due to increasing doping can rapidly increase the superconducting transition temperature of the sample even to the value of $T_{c}^{⋆}∼2T_{c}$ (dense disorder leading to strong inhomogeneity). This phenomenon seems to be characteristic of high-temperature superconductors and superconducting heavy fermions, and recently have received renewed attention. We experimentally documented the stronger lattice stiffening of the inhomogeneous superconducting phase $T_{c}^{⋆}$ in respect to the bulk $T_{c}$ one and proposed a model that explains the $T_{c}^{⋆}>T_{c}$ behavior in the series of nonmagnetic skutterudite-related compounds.

## 1. Introduction

The effect of atomic disorder on superconducting properties has been the cause of intense research, both experimental and theoretical, since the BCS theory [1] explained the mechanism of superconductivity. The earliest understanding was due to Anderson’s theory [2], which predicts a negligible effect of nonmagnetic impurities on the superconducting temperature $T_{c}$, as long as the system remains a metal. At the other extreme, magnetic scatterers in a conventional isotropic superconductor suppress $T_{c}$ according to the Abrikosov–Gor’kov law [3] (see also [4]). Even a small amount of magnetic dopants can drastically reduce the critical temperature of the superconducting state [5,6]. However, later it was documented experimentally and by theory, that the Anderson theorem does not hold true in a strongly disordered nonmagnetic superconducting system. In iron-based superconductors, the nonmagnetic scatterers can also suppress $T_{c}$ at the same fast rate in a two-band $s_{\pm }$ state, and the scattering is purely *interband* in nature [7]. Therefore, observations of an enhancement of the superconducting transition temperature, when the amount of lattice disorder in the superconductor is increased, are particularly interesting. We know of rare examples of disorder-enhanced superconductivity, most of the known behavior of this type has been observed in strongly correlated superconductors (SCS), e.g., PrOs${}_{4}$Sb${}_{12}$ [8,9,10,11,12], CeIrIn${}_{5}$ [13], or CePt${}_{3}$Si [14]. The investigations of the increase in $T_{c}$ of the disordered superconductors is, therefore, important not only for better understanding of the mechanism of superconductivity in the $high-T_{c}$ and SCS materials, but also for identifying the possible applications of these new superconductors. It is worth noting that there are also known examples of superconductivity enhancement due to disorder in high-temperature superconductors, e.g., in Bi${}_{2}$Sr${}_{2}$CaCu${}_{2}$O${}_{8+x}$ [15,16].

Our present studies are focused on skutterudite-related superconductors for which we have documented a similar enhancement of $T_{c}$ caused by disorder. By varying the degree of disorder, we attempt to understand the role of various atomic defects and fluctuations in composition on the superconductivity of these materials. The cubic La${}_{3}M_{4}$Sn${}_{13}$ and Ca${}_{3}$Rh${}_{4}$Sn${}_{13}$, or tetragonal Y${}_{5}$Rh${}_{6}$Sn${}_{18}$ and Lu${}_{5}$Rh${}_{6}$Sn${}_{18}$ quasiskutterudites have been reported as *s*-wave BCS superconductors with atomic-scale disorder, which generates a novel superconducting state with critical temperature $T_{c}^{⋆}$ larger than $T_{c}$ of the bulk remaining phase. The aim of the current report is to summarize the knowledge about the superconductivity of the family of quasiskutterudites mentioned above on the basis of our results already published in Refs. [17,18,19,20,21,22], as well as theoretical modeling of the “*high-temperature*” superconductivity in the disordered phase. In order to supplement the literature data, we present here a microanalysis studies of these materials, and discuss the impact of the degree of disorder on the superconductivity of each system. The research was extended by investigations of the local inhomogeneities found in PrOs${}_{4}$Sb${}_{12}$, and the impact of atomic disorder on the superconductivity of this unique Pr-based material. Based on the spectrum of literature data and our recent published results, we have proposed a phenomenological model that explains the relationships $T_{c}^{⋆}>T_{c}$ and $|\frac{dT_{c}^{⋆}}{dP}|>|\frac{dT_{c}}{dP}|$ due to the greater lattice stiffening of the disordered $T_{c}^{⋆}$ phase. Since the degree of the lattice stiffness is well expressed by the Grüneisen parameter, $\Gamma_{G}$, we found $\Gamma_{G}$ of La${}_{3}$Rh${}_{4}$Sn${}_{13}$ larger for its inhomogeneous *high-temperature* $T_{c}^{⋆}$-phase with respect to the bulk $T_{c}$-state (Section 4.3), which can explain the $T_{c}^{⋆}>T_{c}$ behavior.

We also discuss the known theoretical models describing the temperature dependencies of the upper critical fields $H_{c2}$, obtained experimentally for various skutterudite-related components of the series. Depending on the degree of disorder, we prove the correctness of the Werthamer–Helfand–Hohenberg theory or the percolation model, both of which are considered within the dirty limit of the BCS superconductor.

## 2. Experimental Details

The La${}_{3}M_{4}$Sn${}_{13}$, Ca${}_{3}$Rh${}_{4}$Sn${}_{13}$, Y${}_{5}$Rh${}_{6}$Sn${}_{18}$, and Lu${}_{5}$Rh${}_{6}$Sn${}_{18}$ samples were prepared by arc melting technique. The proper dilute alloys, e.g., La${}_{3}$Ru${}_{4-x}$Co${}_{x}$Sn${}_{13}$, or Y${}_{5-x}$Ca${}_{x}$Rh${}_{6}$Sn${}_{18}$ and others, were prepared by arc melting by diluting nominal compositions of the parent compounds. To ensure homogeneity, each sample was turned over and remelted several times, and annealed at 870 ${}^{\circ }$C for 2 weeks. Single crystals of PrOs${}_{4}$Sb${}_{12}$ were grown by the Sb flux method. All samples were examined by X-ray diffraction analysis and found to be single phase with cubic (La${}_{3}M_{4}$Sn${}_{13}$, Ca${}_{3}$Rh${}_{4}$Sn${}_{13}$, space group P$m\bar{3}n$; PrOs${}_{4}$Sb${}_{12}$, space group I$m\bar{3}$), or tetragonal (Y${}_{5}$Rh${}_{6}$Sn${}_{18}$, Lu${}_{5}$Rh${}_{6}$Sn${}_{18}$, space group I$4_{1}/acd$) structure, respectively. Stoichiometry and homogeneity were checked by the electron microscope technique (scanning microscope JSM-5410). To obtain the surface images and structural properties of nanometer-size crystallites of Y${}_{5}$Rh${}_{6}$Sn${}_{18}$ the transmission electron microscopy observations were obtained with a JEOL high-resolution JEM 3010 microscope (see Ref. [21]). Thermodynamical investigations (specific heat *C*, ac magnetic susceptibility $\chi_{ac}$, magnetization *M*) and resistivity ρ were investigated using a Quantum Design (QD) Physical Properties Measurements System (PPMS) device and QD superconducting quantum interference device (SQUID) magnetometer.

## 3. The Meaning of *Disorder* in the System of Skutterudite-Related La${}_{3}M_{4}$Sn${}_{13}$-Type Superconductors; Enhancing Superconductivity by Atomic Disorder

Investigations of atomic-scale disorder in the quasiskutterudite superconductors in the form of local defects and vacancies, granularity, and the effective increase of disorder by doping have received renewed attention particularly because of observations of enhancing superconductivity in these materials. This phenomenon is particularly interesting in a situation of dirty superconductors, where defects can disturb the pair-breaking strength, which usually leads to a decrease in $T_{c}$. Our most recent studies focus on the family of nonmagnetic cage Remeika phases [23,24], which exhibit evidence of the impact of atomic defects both on their normal-state and enhancement of superconducting properties. An increase of a static disorder by the atomic defects as well as atomic displacements, evidenced in the series of $R_{3}M_{4}$Sn${}_{13}$ or isoelectronic $R_{5}$Rh${}_{6}$Sn${}_{18}$ superconductors (R= Ca, Y, La, Lu), gives the basis for interpreting the increase in $T_{c}$ at the level of the dilute disorder case (cf. Ref. [25]). Doping at a low concentration level can also be considered as an elemental impurity effect. In the case of more inhomogeneous samples, fluctuations both in atomic disorder and composition are also possible and lead to a more significant increase in $T_{c}$ [25,26]. A special case could be a phase separation observed in Y${}_{5-x}$Ca${}_{x}$Rh${}_{6}$Sn${}_{18}$, where the end points: Y${}_{5}$Rh${}_{6}$Sn${}_{18}$ and Ca${}_{3}$Rh${}_{4}$Sn${}_{13}$ are not formed as isostructural compounds [21]. In this case, the main phase of (Y:Ca)${}_{5}$Rh${}_{6}$Sn${}_{18}$-type is formed as a nontrivial structural deformation of the cubic minority phase (Ca:Y)${}_{3}$Rh${}_{4}$Sn${}_{13}$ [21,22]. Both phases have similar stoichiometry (i.e., 3:4:13 and 3.33:4:12, in effect of normalization of the number of Rh to 4 per each formula unit); therefore, one can consider them as the areas with strong stoichiometry fluctuations around the average composition. If the two-phase system Y${}_{5-x}$Ca${}_{x}$Rh${}_{6}$Sn${}_{18}$ for x>1.2 can be approximated by strongly fluctuating inhomogeneities of the sample composition, then it is possible to explain a large difference in $T_{c}^{⋆}-T_{c}∼2$ K on the basis of Gastiasoro and Andersen’s [25] theoretical model in approximation of the presence of strong fluctuations in the composition of the sample.

**Figure 1:**
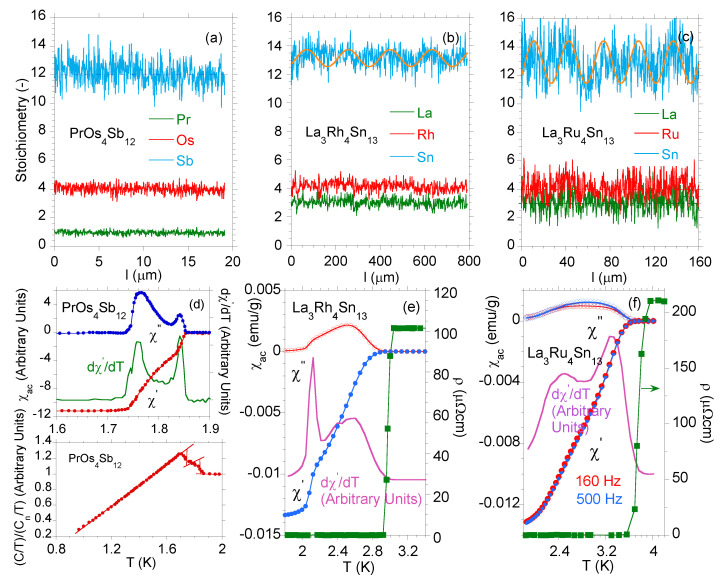
Variation in stoichiometry over the length of the sample: (**a**) for PrOs${}_{4}$Sb${}_{12}$, the correct stoichiometric ratio of Pr:Os:Sb from EDS analysis is 1.00:4.0:12.70, (**b**) for La${}_{3}$Rh${}_{4}$Sn${}_{13}$, the correct stoichiometric ratio of La:Rh:Sn is 3.12:4.0:12.98, (**c**) for La${}_{3}$Ru${}_{4}$Sn${}_{13}$, the correct stoichiometric ratio of La:Ru:Sn is 3.09:4.0:12.78. The yellow curve in panel (**b**,**c**) is a rough approximation of the fluctuations in Sn content around the mean Sn content by $\Lambda \sin(\frac{2\pi }{\Delta l}l)$, where Δl=190 μm for La${}_{3}$Rh${}_{4}$Sn${}_{13}$ and Δl=30 μm for La${}_{3}$Ru${}_{4}$Sn${}_{13}$. Δl expresses a diameter of the extent of strong fluctuations in composition with an amplitude Λ (Λ defines the maximum deviation in sample composition from average Sn content). In approximation of $\frac{2\pi }{\Delta l}\to 0$, the fluctuations in the composition of the sample disappear. Lower panels exhibit the low-temperature $\chi_{ac}\left(T\right)$ data ($\chi_{ac}=\chi^{\prime }+i\chi^{\prime \prime }$) for PrOs${}_{4}$Sb${}_{12}$ (**d**), La${}_{3}$Rh${}_{4}$Sn${}_{13}$ (**e**), and La${}_{3}$Ru${}_{4}$Sn${}_{13}$ (f) (the $\chi_{ac}$ data for PrOs${}_{4}$Sb${}_{12}$ are taken from Ref. [11]). Panel (**d**) also shows the specific heat C(T)/T of PrOs${}_{4}$Sb${}_{12}$, normalized to the value of C/T at T=1.9 K in the normal state of the sample. The reason for this is that the specific heat of various single crystals obtained from the same melting batch shows different values of *C* in the low *T* region due to different Sb contents in PrOs${}_{4}$Sb${}_{12}$ [12]. (**d**–**f**) The perfect diamagnetism of the full Meissner state with $\chi^{\prime }=-1/\left(4\pi d\right)$ for mass density *d* is reached at the lowest temperatures. Panels (**e**,**f**) display the resistivity data (green filled squares) near $T_{c}^{⋆}$.

**Figure 2:**
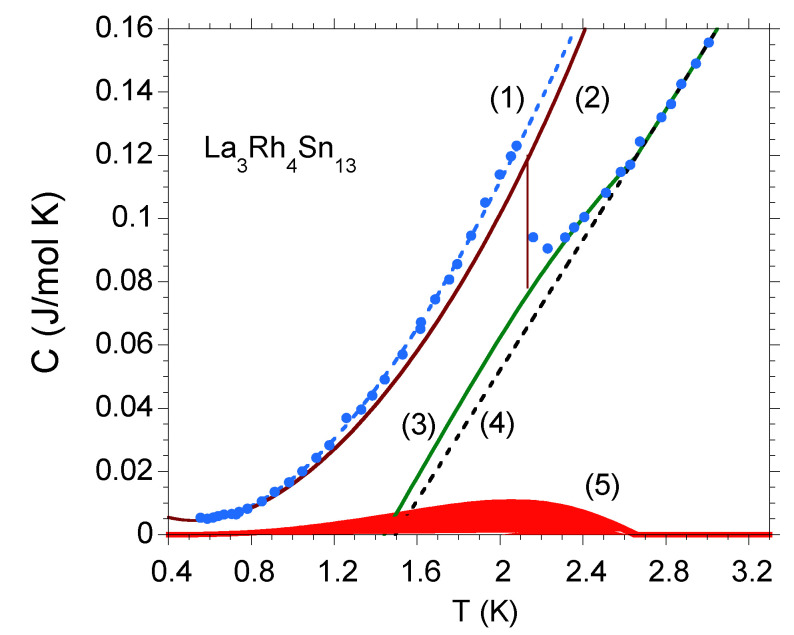
Specific heat C(T) for La${}_{3}$Rh${}_{4}$Sn${}_{13}$ was approximated using the atomic-scale disorder model. $C_{i}$ represents various contributions to C(T), i=1−5. The blue points are the *C* experimental data that are well fitted by the expression $C_{1}\left(T\right)=\gamma_{0}T+\beta T^{3}+A\exp\left[-\Delta \left(0\right)/k_{B}T\right]$ with the electronic specific heat coefficient $\gamma_{0}=6$ mJ/mol K${}^{2}$, lattice contribution with β=6.5 mJ/mol K${}^{4}$, and energy gap Δ(0)=4.2 K at T=0. $C_{5}$ represents the best fit of the Gausian gap distribution f(Δ) with $\Delta_{0}=2.06$ K and D=0.25 K${}^{2}$. $C_{4}\left(T\right)=\gamma_{0}T$ is the electronic contribution to the specific heat. The curve $C_{3}=C_{4}+C_{5}$, $C_{2}$ (red line) was obtained after subtracting the inhomogeneous contribution $C_{5}$ from the experimental data.

Figure 1 shows the evolution of the degree of disorder in the series of various skutterudite-related compounds. Panel (a) displays evidence of nanoscale inhomogeneity as a bulk property of PrOs${}_{4}$Sb${}_{12}$ single crystal over the length scale similar to the coherence length, which is a reason of appearance of the *high temperature inhomogeneous superconducting phase* with characteristic critical temperature $T_{c}^{⋆}=1.84$ K in the *bulk* superconducting state below $T_{c}=1.76$ K (cf. Refs. [8,9,10,11,12]). Panel (b) displays observations either of local atomic disorder and weak fluctuation in composition of La${}_{3}$Rh${}_{4}$Sn${}_{13}$ within ∼190 μm volume fraction, while Figure 1c shows the observation of strong fluctuations in composition in the La${}_{3}$Ru${}_{4}$Sn${}_{13}$ sample area of ∼30 μm. The lower panels compare the real ($\chi^{\prime }$) and imaginary ($\chi^{\prime \prime }$) parts of ac mass magnetic susceptibility $\chi_{ac}$, and derivative $d\chi^{\prime }/dT$, respectively, for single crystalline PrOs${}_{4}$Sb${}_{12}$ (d), La${}_{3}$Rh${}_{4}$Sn${}_{13}$ (e), and La${}_{3}$Ru${}_{4}$Sn${}_{13}$ (f). The double superconducting transitions in the good PrOs${}_{4}$Sb${}_{12}$ single crystal are divided into macroscopically segregated parts: one with distribution of $T_{c}^{⋆}$ and the second with a single superconducting transition at $T_{c}$ which is intrinsic—both transitions are sharp with ΔT≈0.03 K at the respective critical temperature. The Ca${}_{3}$Rh${}_{4}$Sn${}_{13}$ [19] and La${}_{3}$Co${}_{4}$Sn${}_{13}$ [17] superconductors behave similarly, they exhibit sharp transitions at $T_{c}^{⋆}$ and $T_{c}$; however, $T_{c}^{⋆}≅T_{c}$, which suggests that these superconductors are homogeneous with possible nanoscale atomic disorder leading to nanoscale electronic inhomogeneity. However, the maximum value of derivative $d\chi^{\prime }/dT$ assigned to the distribution of the critical temperatures $T_{c}^{⋆}$ in La${}_{3}$Rh${}_{4}$Sn${}_{13}$ (e) is broad with a half width at ΔT≈0.6 K, while that attributed to the transition at $T_{c}$ was observed to be much narrower (ΔT≈0.05 K). The $\chi_{ac}$ data presented in panel (f) show very broad transitions both at $T_{c}^{⋆}$ and $T_{c}$ of La${}_{3}$Ru${}_{4}$Sn${}_{13}$, which correlate with the documented strong atomic disorder and fluctuations in stoichiometry for this sample. The analogous behavior to that, shown in panels (c) and (f) was previously documented for a number of alloys, e.g., for the series of Ca${}_{3}$Rh${}_{4}$Sn${}_{13}$ doped with La or Ce [20], La${}_{3}$Ru${}_{4}$Sn${}_{13}$ doped with Co [18], or Y${}_{5}$Rh${}_{6}$Sn${}_{18}$ doped with Ca [21], all systems are strongly disordered. It is worth noting, that the anomaly at $T_{c}^{⋆}$ marks the onset of an inhomogeneous superconducting phase with spatial distribution of the magnitude of the superconducting energy gaps. Following [17], a simple Gaussian gap distribution $f\left(\Delta \right)\propto \exp\left[-\frac{{\left(\Delta -\Delta_{0}\right)}^{2}}{2D}\right]$ approximates, e.g., the specific heat data at $T_{c}<T<T_{c}^{⋆}$, where $\Delta_{0}$ and variance *D* of the distribution are treated as fitting parameters (see Figure 2). The maximum of the f(Δ) distribution also agrees with the *T*-dependence of $d\chi {}^{\prime }/dT$ and $\chi {}^{\prime \prime }$ maxima in Figure 1e,f.

**Figure 3:**
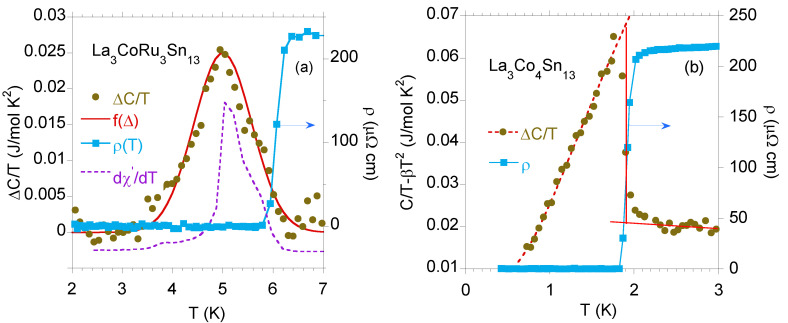
(**a**) The temperature dependence of the specific heat $\frac{\Delta C(T)}{T}$ of La${}_{3}$CoRu${}_{3}$Sn${}_{13}$ was well approximated by function f(Δ) with the following parameters: $\Delta_{0}=5.0$ K and variance D=0.3 K${}^{2}$, where ΔC(T)=C(T,B=0)−C(T,B=5T). The derivative $\frac{d\chi {}^{\prime }}{dT}$ is also shown in arbitrary units. For comparison, panel (**b**) exhibits $\frac{C(T)}{T}$ for La${}_{3}$Co${}_{4}$Sn${}_{13}$ subtracted by the phonon contribution $\beta T^{2}$, β=3.5 mJ/mol K${}^{2}$. The $\frac{\Delta C(T)}{T}$ data are well approximated by expression $\frac{\Delta C(T)}{T}=\gamma_{0}+\frac{A}{T}\exp\left[\frac{-\Delta (0)}{k_{B}T}\right]$ (red dotted line) with the fitting parameters $\gamma_{0}=$ 8 mJ/mol K${}^{2}$ and superconducting energy gap Δ(0)=2.6 K. La${}_{3}$Co${}_{4}$Sn${}_{13}$ does not form the disordered $T_{c}^{⋆}$ phase. Both panels show the resistivity ρ(T) near the superconducting transition temperature. The critical temperature $T_{c}^{⋆}$ [in panel (**a**)] or $T_{c}$ (**b**) is defined as *T* at 50% of the normal-state ρ value.

In the case of strongly inhomogeneous superconductors, the mesoscopic size disorder can be a reason of large modulation of the superconducting gap, which, in consequence, leads to a large transition width to the superconducting state. In this case, both C(T) and $\chi_{ac}\left(T\right)$ show a weak and broad transition with the maximum in *C* or $\chi_{ac}$ data at $T_{c}^{⋆}$, which covers the transition at $T_{c}$, and is well described by the function f(Δ). For example, Figure 3a shows the anomalies in C(T)/T and $\chi_{ac}\left(T\right)$ data, seen over a wide temperature range below $T_{c}^{⋆}$, where the critical temperature was obtained from the resistivity ρ(T) data. In such a strongly disturbed system [panel (a)], a type II metal–superconductor transition is broad and weakly visible, in contrast to that, measured for the La${}_{3}$Co${}_{4}$Sn${}_{13}$ BCS superconductor [as shown in panel (b)].

**Figure 4:**
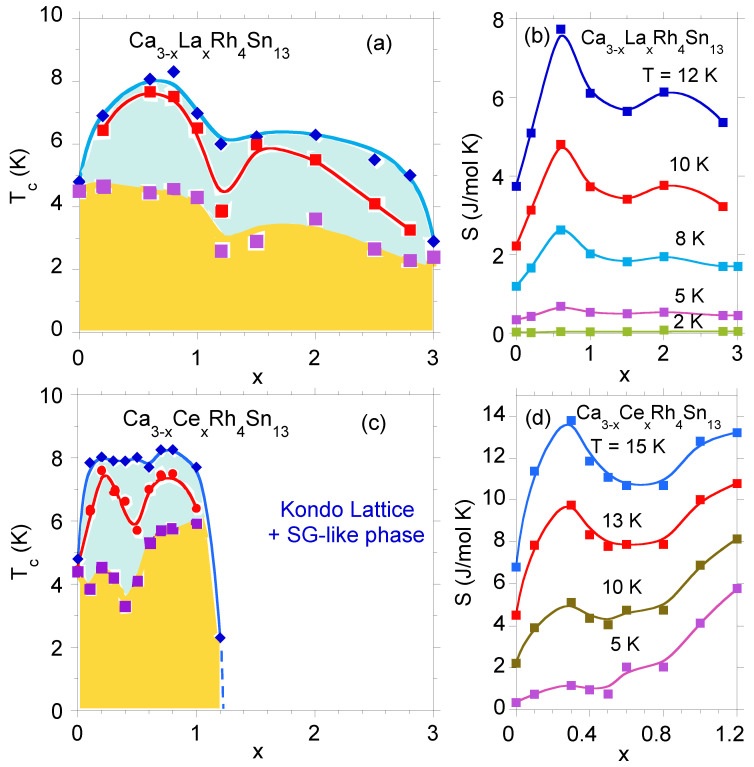
T−x phase diagram for Ca${}_{3-x}$La${}_{x}$Rh${}_{4}$Sn${}_{13}$ (panel **a**) and Ca${}_{3-x}$Ce${}_{x}$Rh${}_{4}$Sn${}_{13}$ (**c**). The blue color shows the area of the *high $T_{c}^{⋆}$* phase, the blue line indicates the beginning of the transition from the normal to SC phase, while the red line shows the temperature of the maximum of the f(Δ) function. The yellow color shows the area of the *bulk*$T_{c}$ phase. Panels (**b**,**d**) show the entropy isotherms as a function of *x* for Ca${}_{3-x}$La${}_{x}$Rh${}_{4}$Sn${}_{13}$ and Ca${}_{3-x}$Ce${}_{x}$Rh${}_{4}$Sn${}_{13}$, respectively.

The T−x diagram shown in Figure 4 clearly indicates the separated superconducting $T_{c}$ and $T_{c}^{⋆}$ phases for Ca${}_{3}$Rh${}_{4}$Sn${}_{13}$, when it is doped with La and Ce. An increase in atomic disorder due to increased doping enhances the separation of $T_{c}$ and $T_{c}^{⋆}$, which is well reflected by the entropy isotherms $S_{T}\left(x\right)$ shown in panels (b) and (d), respectively (for details see Ref. [20]). This is a rare example where atomic disorder as a result of doping, acting as perturbation of the lattice periodicity, enhances superconductivity. In this case, both pristine compounds crystallize in the same cubic structure P$m\bar{3}n$, thus the disorder can be treated here as a concentration variable *x*. At the concentration level 0<x<3, the increased doping is reflected by a smooth change in the lattice parameters and volume of the unit cell as a function of *x* [20]; hence, the doping can be considered as an elemental impurity effect, giving the basis for interpreting the increase in $T_{c}$ at each level of the disorder. Here, one should note, that in the case of the series of compounds with end-points, which are not formed as isostructural compounds, the systematic replacement of the atoms no longer simply reflects “disorder” as a variable dependent of the concentration *x*; this is the case for the Y${}_{5-x}$Ca${}_{x}$Rh${}_{6}$Sn${}_{18}$ series (cf. [21]).

Finally, we want to explain why for some skutterudites and quasiskutterudites, the literature data refer to different values of $T_{c}$, e.g., recent results revealed an intrinsic superconducting transition at 3.8 K [27] for La${}_{3}$Rh${}_{4}$Sn${}_{13}$, or 8.4 K for Ca${}_{3}$Rh${}_{4}$Sn${}_{13}$ [28] (both samples were obtained as single crystals) instead of 2.28 K [17] or 4.8 K [19] obtained for respective polycrystalline samples. It was reported for Ca${}_{3}$Rh${}_{4}$Sn${}_{13}$ [28,29], that antisite defects Ca-Sn1 generated at high temperatures in the melting process and then frozen-in by quenching to room temperature are responsible for the strong lowering of $T_{c}$ and reduction in the unit cell volume of this superconductor. Following this, the value of $T_{c}=4.8$ K for a polycrystalline Ca${}_{3}$Rh${}_{4}$Sn${}_{13}$ sample rapidly quenched during the arc melting process can be expected, as was discussed in details in Ref. [19]. This change in $T_{c}$ can also be explained by simple phenomenology, when considering Ca${}_{3}$Rh${}_{4}$Sn${}_{13}$ under different heat treatments, a linear relationship was observed between its critical temperature and the lattice volume. Our previous *ab initio* calculations documented a linear decrease of DOS for Ca${}_{3}$Rh${}_{4}$Sn${}_{13}$ at $\epsilon_{F}$ with decreasing sample volume, as a result of rapid quenching. The calculated change of DOS is a reason for the decrease in $T_{c}$ and quantitatively determines the reduction of $T_{c}=8.4$ K of a single crystalline sample to the value ∼4.8 K for the polycrystalline one. This behavior follows from the BCS equation [1]

(1) $$T_{c}=1.14\langle \omega \rangle \exp\left[-1/\left(N\left(\epsilon_{F}\right)U\right)\right],$$

where $N(\epsilon_{F})$ is the DOS at the Fermi energy in states per eV and per spin and $\langle \omega^{2}\rangle$ is an average of the square of the phonon frequency ($\langle \omega \rangle ∼\theta_{D}/1.2$), and the expression [30]

(2) $$N\left(\epsilon_{F}\right)U\to \frac{\lambda -\mu^{⋆}}{1+\lambda }.$$

Parameter $\mu^{*}$ is the Coulomb pseudopotential of Morel and Anderson [31],

(3) $$\mu^{*}=\frac{N(\epsilon_{F})U}{1+N\left(\epsilon_{F}\right)U\ln\left(E_{B}/\omega_{0}\right)},$$

and electron–phonon coupling parameter [32,33]

(4) $$\lambda =\frac{N\left(\epsilon_{F}\right)\langle I^{2}\rangle }{M\langle \omega^{2}\rangle }.$$

$\langle I^{2}\rangle$ is the square of the electronic matrix element of electron–phonon interactions averaged over the Fermi surface, $E_{B}$ is the electronic bandwidth, and $\omega_{0}$ is the maximum phonon frequency ($\omega_{0}>\theta_{D}$), and $\theta_{D}$ is the Debye temperature. A similar mechanism can explain variations in $T_{c}$ reported in the literature data for La${}_{3}$Rh${}_{4}$Sn${}_{13}$ and other superconducting quasiskutterudites (note, we also obtained the single crystals of La${}_{3}$Rh${}_{4}$Sn${}_{13}$ with $T_{c}^{⋆}=3.76$ K and $T_{c}=2.85$ K by the flax method).

## 4. Superconductivity in the Presence of Atomic Disorder; Dirty Limit

### 4.1. The Temperature Dependence of the Critical Field $H_{c2}$—Modeling on the Base of the Werthamer-Helfand-Hohenberg Theory

The upper critical field $H_{c2}$ in a dirty superconductor with a mean free path l≪ξ that can be explained by the Werthamer–Helfand–Hohenberg (WHH) [34,35,36] or Maki-de Gennes [37,38,39] theories. This theoretical model predicts a linear change of $H_{c2}$ with *T* near the critical temperature $T_{c}$, and

(5) $$H_{c2}\left(0\right)=-0.69T_{c}{\left.\frac{dH_{c2}}{dT}\right|}_{T=T_{c}}.$$

The WHH formula [Equation (5)] for a type-II dirty one-gap superconductor allows for the zero temperature upper critical field $H_{c2}\left(0\right)$ to be estimated, while the $H_{c2}\left(T_{c}\right)$ curve in the whole superconducting range $0-T_{c}$ can be calculated using the di–gamma function ψ, as was proposed by Werthamer et al. [34] [see also Equations (6) and (7)]. The issue is more complicated in the case of the multiband model. For a two-band dirty superconductor, $H_{c2}\left(T\right)$ can be calculated from the theory of Gurevich [40], which is obtained adapting the Eilenberger and Usadel equations to the case of a two-band dirty superconductor,

(6) $$a_{0}\left[\ln t+U(h)\right]\left[\ln t+U(\eta h)\right]+a_{2}\left[\ln t+U(\eta h)\right]+a_{1}\left[\ln t+U(h)\right]=0,$$

where $U\left(x\right)\equiv \psi \left(x+1/2\right)-\psi \left(1/2\right)$, ψ(…) is the di–gamma function, $t=T/T_{c}$, *h* is reduced magnetic field defined as $h=H_{c2}D_{1}/2\Phi_{0}T$, $D_{1}$ is the band diffusivity, $\eta =D_{2}/D_{1}$. The parameters $a_{0,1,2}$ are expressed by the intra- and interband BCS superconducting coupling constants $\lambda_{11},\;\lambda_{22},\;\lambda_{12}$ and $\lambda_{21}$, respectively. In the case of a one-band model, Equation (6) reduces to the standard Maki-de Gennes equation for $H_{c2}$

(7) $$\ln\frac{1}{t}=U\left(h\right).$$

**Figure 5:**
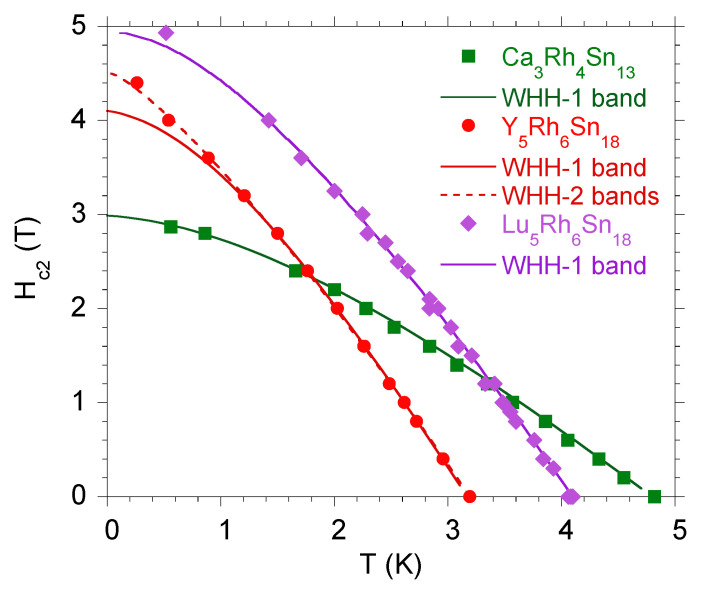
The upper critical field $H_{c2}$ in the H−T plane for Ca${}_{3}$Rh${}_{4}$Sn${}_{13}$, Y${}_{5}$Rh${}_{6}$Sn${}_{18}$, and Lu${}_{5}$Rh${}_{6}$Sn${}_{18}$ approximated by the one-band (solid line) and two-band (dotted line) Werthamer–Helfand–Hohenberg (WHH) model.

For most of the investigated skutterudite-related compounds, the ξ(0) and l(0) parameters determined in the framework of Ginzburg–Landau–Abricosov–Gorkov theory of the type-II superconductors [35,41] obey the relation l≪ξ and a one-band WHH theoretical model usually fits the data on the H−T diagram well, as is shown for Ca${}_{3}$Rh${}_{4}$Sn${}_{13}$ ($H_{c2}\left(0\right)=3.1$ T) and Lu${}_{5}$Rh${}_{6}$Sn${}_{18}$ ($H_{c2}\left(0\right)=5.2$ T) in Figure 5. The respective values of $\frac{dH_{c2}}{dT}$ at $T_{c}$ used for fitting the WHH are listed in Table 1. However, there are known exceptions when $H_{c2}\left(T\right)$ can be affected by the presence of two bands, this is a case of Y${}_{5}$Rh${}_{6}$Sn${}_{17}$. As can be seen in Figure 5, the one-gap WHH model failed to describe its $H_{c2}\left(T\right)$ dependence. The multi-band WHH model also effectively describes the upper critical field in the *H-T* diagram for Ca${}_{3-x}R_{x}$Rh${}_{4}$Sn${}_{13}$ alloys, when Ca${}_{3}$Rh${}_{4}$Sn${}_{13}$ is doped with La or Ce (as shown in Figure 7 in Ref. [20]), which results in their more complicated electronic structure with calculated various electronic states of La/Ce dopants, located near the Fermi level . In the presence of lattice disorder and the effect of breaking the lattice periodicity due to doping, an alternative for describing the H−T behaviors of these alloys could be the percolation model, which also effectively describes the upper critical field of the components *x* of Ca${}_{3}$Rh${}_{4}$Sn${}_{13}$ (will be discussed in the next section).

### 4.2. $H_{c2}$ within the Percolation Modeling for Strongly Inhomogeneous Superconductors; the Case of La${}_{3}$Rh${}_{4}$Sn${}_{13}$

The WHH theory, even in the multi-band version, can be insufficient to explain the temperature dependence of the upper critical filed. It is based on a dirty-limit approximation and may need to be complemented by taking into account the effect of the disorder-induced inhomogeneous carrier distribution. The carrier concentration in these systems is a few orders of magnitude smaller than the typical values for metals [21,42]. Thus, weaker screening of charged impurities can lead to fluctuations of the local chemical potential and induce spatial fluctuations of the superconducting order parameter Δ=Δ(r) [43]. As a result, regions of space where the amplitude of Δ is large are surrounded by regions with relatively small Δ. For weak disorder, increasing the temperature or magnetic field suppresses superconductivity in a BCS-like manner in the entire sample, whereas stronger disorder can lead to superconducting “islands” embedded in normal or even insulating regions. Different superconducting regions usually have different local critical temperatures $T_{c}\left(r\right)$ (this is well documented for the series of Ca${}_{3-x}$La${}_{x}$Rh${}_{4}$Sn${}_{13}$ and Ca${}_{3-x}$Ce${}_{x}$Rh${}_{4}$Sn${}_{13}$ compounds, as shown in Figure 4) and the macroscopic critical temperature depends not only on microscopic superconducting properties of pure materials, but also on the topology of the grain system. The superconducting transition occurs when a percolation path is formed across the system.

Below, we propose a simple model that is able to reproduce the temperature dependence of $H_{c2}$ in La${}_{3}$Rh${}_{4}$Sn${}_{13}$ and other similar systems, where the single-band WHH theory cannot explain the experimental data. By adjusting model parameters, the model can describe a general positive curvature of $H_{c2}$.

**Figure 6:**
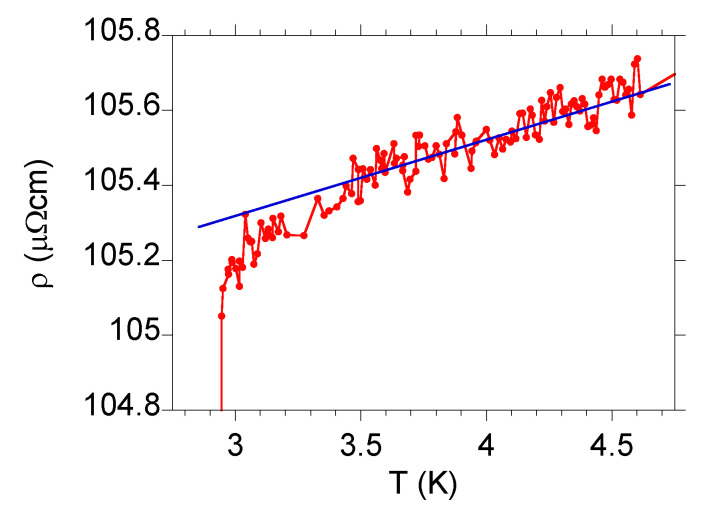
Resistivity of La${}_{3}$Rh${}_{4}$Sn${}_{13}$ near the superconducting transition. The blue solid line indicates a linear decrease of ρ when the temperature decreases. Note, that close to $T_{c}^{*}$, the resistivity decreases faster than linearly, which may indicate formation of disconnected superconducting regions. These give the superconducting transition a percolation path upon forming.

We assume that the inhomogeneous system can be described by the random resistor network (RRN) model [44,45]. The RRN model uses the percolation theory for the hopping conductivity that is based on the notion that the transport equations can be cast into the form of an equivalent RRN. When the temperature approaches the critical value (from above) in a inhomogeneous superconductor, more and more superconducting regions are formed. In the RNN model, it is translated into an increasing number of resistors with zero resistivity. As long as the zero resistivity elements do not form a continuous path across the system, the sample remains in the normal state, but possibly with decreasing resistivity. By calculating the resistance of the RNN, the normal–state transport properties can be determined. However, in the case of La${}_{3}$Rh${}_{4}$Sn${}_{13}$, the normal–state resistivity does not change significantly when temperature approaches $T_{c}$, as shown in Figure 6.

**Figure 7:**
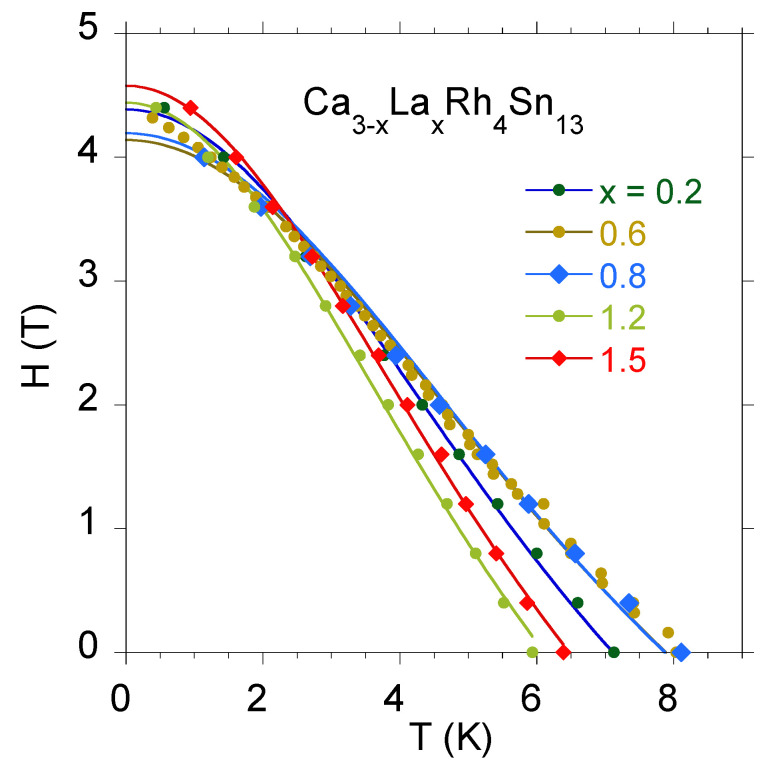
Temperature dependence of the upper critical field for doped La${}_{3}$Rh${}_{4}$Sn${}_{13}$. Note the negative correlation between $H_{c2}\left(T=0\right)$ and $T_{c}\left(H=0\right)$. The continuous lines show the best fit of Ginzburg–Landau theory to experimental data, $H_{c2}\left(T\right)=H_{c2}\left(0\right)\frac{1-t^{2}}{1+t^{2}}$.

This means that in the corresponding RNN model, the resistance of non-superconducting resistors should be large independently of how far the temperature is from $T_{c}$. Therefore, and because we are not interested in the normal state properties, we simply assume that every resistor is in one of two possible states: perfectly conducting or perfectly insulating. Since the resistors represent mesoscopic regions, their state (superconducting or insulating) depends on the temperature and magnetic field. The inhomogeneity of the system leads to variation of the properties of different regions so that we can assign them to be different *local critical temperature* $T_{c}\left(H=0;\;r\right)$. We also assume that within a single mesoscopic region, the single-band WHH theory can be applied. Therefore, we can also introduce *local upper critical field*$H_{c2}\left(r\right)$ given by the solution of Equation (7). The local critical temperature is continuously spread over some range, but for the sake of clarity, let us define only three characteristic local critical temperatures $T_{c}^{\left(1\right)},T_{c}^{\left(2\right)}$ and $T_{c}^{\left(3\right)}$ ($T_{c}^{\left(1\right)}<T_{c}^{\left(2\right)}<T_{c}^{\left(3\right)}$) and three corresponding zero temperature values of $H_{c2}\left(r\right)$: $H_{c2}^{\left(1\right)},H_{c2}^{\left(2\right)}$ and $H_{c2}^{\left(3\right)}$. $T_{c}^{\left(i\right)}$ is defined as the temperature at which the superconducting regions characterized by the upper critical field $H_{c2}^{\left(i\right)}$ form a percolation path across the sample. We assume a linear dependence between $H_{c2}^{\left(i\right)}$ and $T_{c}^{\left(i\right)}$, $H_{c2}^{\left(i\right)}=aT_{c}^{\left(i\right)}+b$, where a<0. This simple form turns out to give a perfect agreement of model predictions with experimental data for La${}_{3}$Rh${}_{4}$Sn${}_{13}$. Different mesoscopic regions may differ in composition, which means that the relation between their parameters can be inferred from the relation between macroscopic values of $H_{c2}$ and $T_{c}$ for systems with different amounts of doping. Such results for Ca-doped La${}_{3}$Rh${}_{4}$Sn${}_{13}$ can be seen in Figure 7.

This behavior is not very uncommon, e.g., similar dependence for nanoscale-SiC doping of MgB${}_{2}$ has been reported [46]. The negative correlation between disorder-induced changes of $H_{c2}^{\left(i\right)}$ and $T_{c}^{\left(i\right)}$ has also been demonstrated within the Ginzburg–Landau theory [47]. Foremost, the sign of the correlation is demonstrated by a direct comparison of the predictions of the model with our data for La${}_{3}$Rh${}_{4}$Sn${}_{13}$.

**Figure 8:**
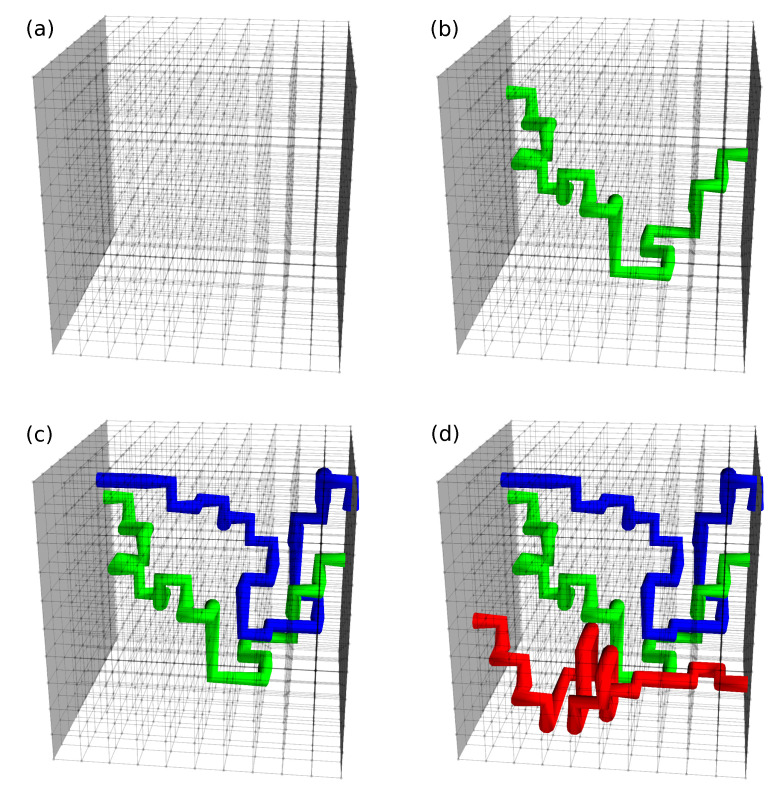
Illustration of percolation path formation when temperature *T* is decreasing, $T>T_{c}^{\left(3\right)}$ (**a**), $T_{c}^{\left(2\right)}<T<T_{c}^{\left(3\right)}$ (**b**), $T_{c}^{\left(1\right)}<T<T_{c}^{\left(2\right)}$ (**c**), and $T<T_{c}^{\left(1\right)}$ (**d**). The green, blue, and red lines represent connected superconducting regions characterized by parameters $\left(T_{c}^{\left(3\right)},H_{c2}^{\left(3\right)}\right),\;\left(T_{c}^{\left(2\right)},H_{c2}^{\left(2\right)}\right)$, and $\left(T_{c}^{\left(1\right)},H_{c2}^{\left(1\right)}\right)$, respectively.

**Figure 9:**
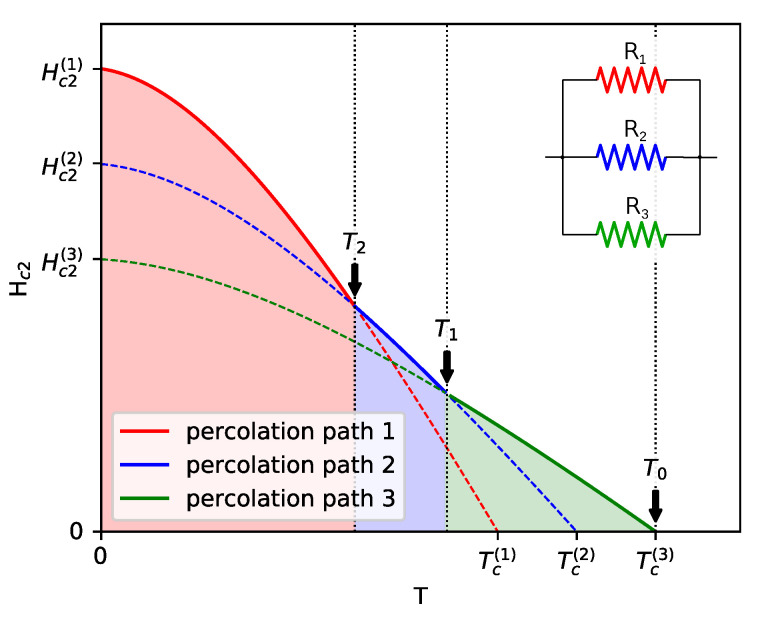
Phase diagram in the T−H plane obtained for the model described in the text. The dashed lines represent solutions of the WHH equations, the solid sections show the macroscopic upper critical field, i.e., the field below which there exists at least one percolation path across the sample. The inset shows an equivalent circuit diagram, where resistance $R_{i}$ is zero for a field smaller than the corresponding solution of the WHH equation [$H<H_{c2}^{\left(i\right)}\left(T\right)$] and infinity otherwise. The green area is a region where only $R_{3}=0$, whereas $R_{2}$ and $R_{1}$ remain finite, which corresponds to the situation depicted in Figure 8b. In the entire blue area $R_{2}=0$, but below the green dashed line, $R_{3}=0$. However, since for temperatures between $T_{1}$ and $T_{2}$ we have $H_{c2}^{\left(2\right)}\left(T\right)>H_{c2}^{\left(3\right)}\left(T\right)$, the upper critical field in this temperature range is determined by resistance $R_{2}$. Similarly, in the entire red area $R_{1}=0$, whereas $R_{2}$ and $R_{3}$ vanish for $H<H_{c2}^{\left(2\right)}\left(T\right)$ and $H<H_{c2}^{\left(3\right)}\left(T\right)$, respectively, i.e., below the blue and green dashed lines.

Figure 8 demonstrates the process of activating different percolation paths when the temperature is decreasing. The resulting temperature dependence of $H_{c2}$ is shown in in Figure 9. The inset shows the corresponding circuit diagram that explains the electric transport measurements.

For temperatures above $T_{c}^{\left(3\right)}$, superconducting regions can exist in the sample, but they are separated and electrical measurements show finite resistance. This situation is depicted in Figure 8a. In the circuit diagram, resistors $R_{1}$, $R_{2}$, and $R_{3}$ have infinite values.

At $T_{c}^{\left(3\right)}$, regions with the zero temperature upper critical field $H_{c2}^{\left(3\right)}$ connect to form a percolation path, shown by the green line in Figure 8b. Resistances $R_{1}$ and $R_{2}$ remain infinite, but $R_{3}$ is given by

(8) $$R_{3}=\left\{\begin{array}{cc} 0\; & if\;\;H<H_{c2}^{\left(3\right)}\left(T\right),\\ \infty \; & otherwise, \end{array}\right.$$

where $H_{c2}^{\left(3\right)}\left(T\right)$ is the solution of Equation (7) for $H_{c2}\left(T=0\right)=H_{c2}^{\left(3\right)}$, shown by the solid green line in Figure 9. When the temperature reaches $T_{c}^{\left(2\right)}$ at zero field, a new percolation path is formed by connecting regions characterized by $H_{c2}\left(T=0\right)=H_{c2}^{\left(2\right)}$, as shown by the blue line in Figure 8c. At finite but weak magnetic field *H*, both percolation paths exist, with $R_{2}$ given by

(9) $$R_{2}=\left\{\begin{array}{cc} 0\; & if\;\;H<H_{c2}^{\left(2\right)}\left(T\right),\\ \infty \; & otherwise. \end{array}\right.$$

However, in this temperature range $H_{c2}^{\left(3\right)}>H_{c2}^{\left(2\right)}$ and with increasing magnetic field, the “blue” path is destroyed as the first one, so the upper critical field is determined entirely by $H_{c2}^{\left(3\right)}$. As shown in Figure 9, such a situation holds up to $T=T_{1}$, above which $H_{c2}^{\left(3\right)}<H_{c2}^{\left(2\right)}$ and the actual $H_{c2}$ is defined by $H_{c2}^{\left(2\right)}$. Below $T_{c}^{\left(1\right)}$ the third percolation path, shown by the red line in Figure 8d, is formed by connecting regions with $H_{c2}\left(T=0\right)=H_{c2}^{\left(1\right)}$ and the resistance $R_{3}$ drops to zero for $H<H_{c2}\left(T\right)$. Then, when the temperature crosses $T_{2}$, $H_{c2}^{\left(1\right)}$ becomes the upper critical field for the entire sample.

It is reasonable to assume that in real systems, the temperatures at which different percolation paths are formed has a continuous distribution. Therefore, in order to compare the model with experimental data for La${}_{3}$Rh${}_{4}$Sn${}_{13}$, we assumed 10 values of $T_{c}^{\left(i\right)}$ uniformly distributed between some limiting values $T_{c}$ and $T_{c}^{*}$. The values of corresponding $H_{c2}^{\left(i\right)}$ have been calculated as $H_{c2}^{\left(i\right)}=aT_{c}^{\left(i\right)}+b$ with parameters *a* and *b* have been determined by fitting to the experimental data.

**Figure 10:**
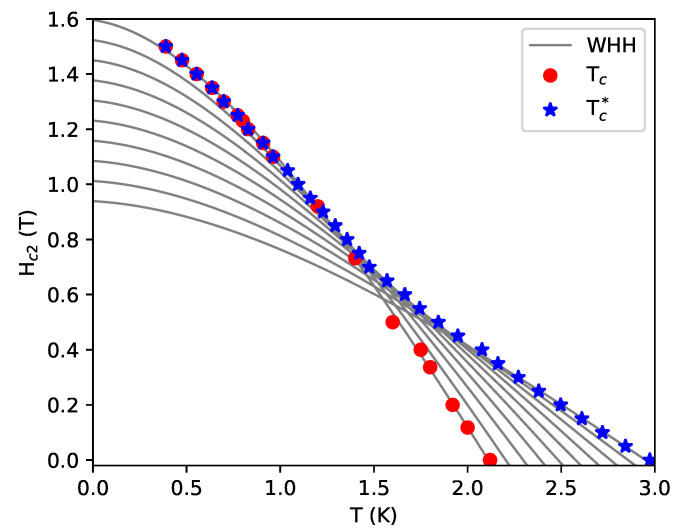
The upper critical field for La${}_{3}$Rh${}_{4}$Sn${}_{13}$ compared with results from the percolation model. The blue stars represent resistivity measurements ($T_{c}^{*})$ and the red circles were determined from heat capacity measurements. The gray lines show WHH solutions under the assumption that $H_{c2}\left(T=0\right)=aT_{c}\left(H=0\right)+b$, with a<0. Parameters *a* and *b* were fitted to reproduce the resistive superconducting transition.

Figure 10 shows a comparison of the upper critical field obtained within the framework of the proposed above model and experimental data for La${}_{3}$Rh${}_{4}$Sn${}_{13}$.

The transition observed in resistivity ($T_{c}^{*})$ is marked by blue stars and the one observed in the heat capacity ($T_{c})$ by red circles. Since percolation is related to electronic transport throughout the sample, the model parameters *a* and *b* were fitted to the observed resistive superconducting transition. One can observe almost perfect agreement. There is still, however, the question about the discrepancy between $T_{c}$ and $T_{c}^{*}$. It can be understood if one assumes that the percolation paths are narrow, quasi-one-dimensional objects. In this case, pairing is not affected by the orbital effects of magnetic field and for H>0, this kind of superconductivity can survive up to higher temperatures ($T_{c}^{*}$) than bulk superconductivity. However, since the superconducting fraction of the volume of the sample is very small, transition to this state is not observed in the heat capacity nor in magnetic susceptibility measurements. At lower temperatures, pairing is strong enough to repel the magnetic field and a transition to bulk superconductivity is observed at $T_{c}$.

The percolation model almost perfectly reproduces the temperature dependence of $H_{c2}$ for La${}_{3}$Rh${}_{4}$Sn${}_{13}$. This suggests that in this compound, the disorder scattering needs to be accompanied by the effect of spatial carrier fluctuations to properly describe the unconventional shape of the critical field. It explains why the WHH theory alone is not sufficient to describe the magnetic properties of this system.

### 4.3. Phenomenology

**Figure 11:**
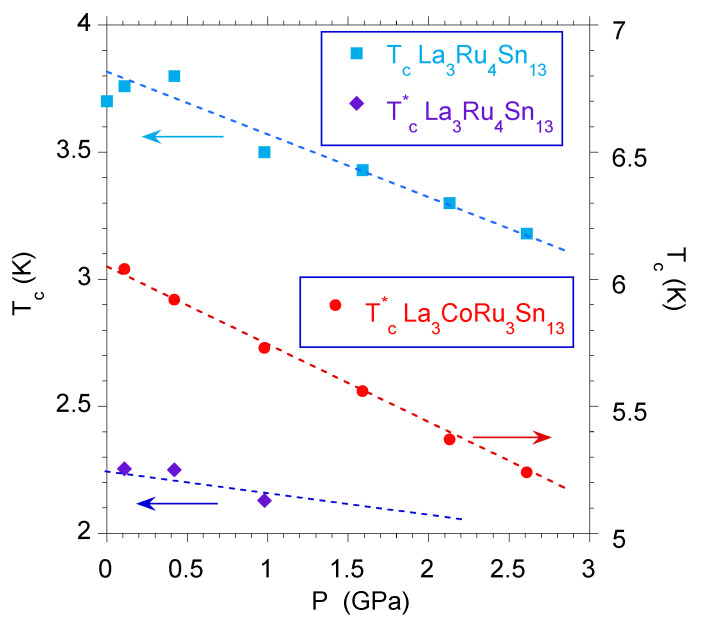
Critical temperatures $T_{c}$ and $T_{c}^{⋆}$ vs. *P* for La${}_{3}$Ru${}_{4}$Sn${}_{13}$. The derivatives equal $\frac{dT_{c}}{dP}=-0.03$ K/GPa and $\frac{dT_{c}^{⋆}}{dP}=-0.24$ K/GPa, respectively. The critical temperatures were obtained from the resistivity under applied pressure at 50% of the normal state value. For comparison, the $T_{c}^{⋆}$ vs. *P* data are also shown for the doped La${}_{3}$Ru${}_{3}$CoSn${}_{13}$ sample ($\frac{dT_{c}^{⋆}}{dP}=-0.32$ K/GPa). The $T_{c}$ vs. *P* data were taken from Ref. [18].

The investigations under external pressure are very useful for modeling the mechanism of superconductivity, especially in strongly disordered materials. Most of the known superconductors exhibit a decrease in $T_{c}$ with an increase in the applied pressure. At the same time, the increase in pressure stabilizes the structural properties of the disordered system by mitigating in part the inhomogeneity of the sample, in consequence $T_{c}^{⋆}$ is expected to also decrease with pressure (see Figure 11). The evidence of this is shown in Refs. [18,19,20,21]. Simultaneously, we documented experimentally, that the pressure coefficients $|\frac{dT_{c}^{⋆}}{dP}|$ are observed as being larger than those of $T_{c}$ (cf. Figure 11), which can be explained on the basis of the Eliashberg theory of strong-coupling superconductivity.

Namely, for all known quasiscutterudite compounds, the electron–phonon coupling parameter $\lambda^{⋆}$ obtained for the inhomogeneous $T_{c}^{⋆}$ superconducting phase is in each case larger than λ of the respective bulk $T_{c}$ superconucting state (cf. Table 1). In Equation (4) $\mu^{⋆}$ and $\langle I^{2}\rangle$ are weakly pressure-dependent (see [21]); therefore, the $\frac{dT_{c}^{⋆}}{dP}$ comes from $\theta_{D}$ and $2N(\epsilon_{F})$, while the *P*-dependence of the Debye temperature is defined by the Grüneisen parameter $\Gamma_{G}=-\frac{d\ln\theta_{D}}{d\ln V}$, which provides information about the lattice stiffening. Our previous data suggest a larger $\Gamma_{G}$ for the disordered superconducting $T_{c}^{⋆}$ phase with respect to the $T_{c}$ one. To calculate λs and $\lambda^{⋆}$s, as listed in Table 1, we used the expression

(10) $$N\left(\epsilon_{F}\right)U=\frac{-\left[2+\lambda \left(1-x\right)\right]+{\left[\lambda^{2}{\left(1+x\right)}^{2}+4\lambda +4\right]}^{1/2}}{2x(1+\lambda )},$$

by combining Equations (2) and (3), where $x=\ln(E_{B}/\omega_{0})$, and $E_{B}∼4.5$ eV is a calculated conduction band width. Equation (1) allows for the calculation of the *experimental* value of $N(\epsilon_{F})U$ and [$N(\epsilon_{F})U$]${}^{⋆}$ for the $T_{c}$ and $T_{c}^{⋆}$ phases, respectively, while Equation (10) gives the λ-dependent variable $N(\epsilon_{F})U$. This self-consistent procedure allowed for the calculation of the λs listed in Table 1 for the best agreement between calculated and experimentally obtained $N(\epsilon_{F})U$.

It is worth noting that the following relationship is always true—$\lambda^{⋆}>\lambda$. In the case of strongly disordered La${}_{3}$Ru${}_{4}$Sn${}_{13}$, or La${}_{3}$Ru${}_{3}$CoSn${}_{13}$ and the Ca${}_{3-x}R_{x}$Ru${}_{4}$Sn${}_{13}$ doped superconductors, $\Delta \lambda =\lambda^{⋆}-\lambda ∼0.1$ was the largest value obtained. We, therefore, analyzed the Grüneisen parameter for La${}_{3}$Ru${}_{4}$Sn${}_{13}$, which represents the series of strongly disordered superconducting quasiskutterudites listed in Table 1, to confirm the hypothesis about different lattice stiffening of the $T_{c}$ and $T_{c}^{⋆}$ superconducting phases. For calculations, we used the expression [30,48]

(11) $$\frac{d\ln[N\left(\epsilon_{F}\right)U]}{d\ln V}\equiv \phi =\left(2\Gamma_{G}-\frac{4}{3}\right)\frac{\lambda }{1+\lambda }\frac{1+\mu^{⋆}}{\lambda -\mu^{⋆}}.$$

For La${}_{3}$Ru${}_{4}$Sn${}_{13}$ϕ=1.43 and $\mu^{⋆}=0.2$ from the McMillan relationship

(12) $$T_{c}=\frac{\theta_{D}}{1.45}\exp\left\{\frac{-1.04(1+\lambda )}{\lambda -\mu^{*}\left(1+0.62\lambda \right)}\right\}.$$

Then, the expression (11) gives $\Gamma_{G}=1.10$ and $\Gamma_{G}^{⋆}=1.23$, for $T_{c}$ and $T_{c}^{⋆}$ phases, respectively. The relation $\Gamma_{G}^{⋆}>\Gamma_{G}$ also explains the experimental observations $|dT_{c}^{⋆}/dP|>|dT_{c}/dP|$ measured for all superconductors listed in Table 1. One can generalize that the relationship between $\Gamma_{G}$ and $T_{c}$ can be extended for all disordered skutterudite-related superconductors (see, e.g., Y${}_{5}$Rh${}_{6}$Sn${}_{18}$ doped with Ca [21]) and the filled skutterudite PrOs${}_{4}$Sb${}_{12}$ superconductor [49]. Here, one can note one of the most interesting results for the single crystal of PrOs${}_{4}$Sb${}_{12}$, namely the observation of two various superconducting transitions at $T_{c}$ and $T_{c}^{⋆}$, and the measured value of $|\frac{dT_{c}^{⋆}}{dP}|$, which is ∼20% larger than $|\frac{dT_{c}}{dP}|$ [49].

## 5. Conclusions

We point out the unique behavior observed for the skutterudite-related compounds whereby lattice disorder enhances the superconducting transition temperature $T_{c}$ to $T_{c}^{⋆}$, where $T_{c}^{⋆}>T_{c}$. It has been shown that their superconducting transition temperature $T_{c}$ depends on the degree of atomic disorder in the system, and that $T_{c}$ increases with random disorder. The reasons for the observed behavior are both the atomic disorder on the nanoscale and the fluctuations in composition in the μm area of the sample, the last one caused a significant increase in $T_{c}$. In both cases, the observed phenomenon can be qualitatively described by the Gastiasoro and Andersen [25] theoretical approach. In a series of our previous reports, we proposed a phenomenological model to explain the increase in $T_{c}$ by the different stiffness of the bulk and the inhomogeneous *high-temperature $T_{c}^{⋆}$* superconducting phases. From BCS theory, the critical temperature $T_{c}$ first of all depends on the value of $\theta_{D}$ and $2N(\epsilon_{F})$, while the pressure dependence of $\theta_{D}$ giving the Grüneisen parameter $\Gamma_{G}=-d\ln\theta_{D}/d\ln V$, defines the lattice stiffening. Our data obtained for various quasiskutterudite samples suggest a larger $\Gamma_{G}^{⋆}$ for the inhomogeneous superconducting phase with respect to the bulk effect below $T_{c}$ (cf. Ref. [21]). La${}_{3}$Ru${}_{4}$Sn${}_{13}$ is a good sample for such investigations, because of the presence of two, well separated $T_{c}$ and $T_{c}^{⋆}$ superconducting states. The experimental data combined with the DFT results allowed for the Grüneisen parameter to be calculated for both its superconducting states and found the relation $\Gamma_{G}^{⋆}>\Gamma_{G}$ between them, which well supports the hypothesis of the stiffening effect on the increase of $T_{c}$.

## Figures and Tables

**Table 1:** The parameters characterizing the superconducting samples. The electronic specific heat $\gamma_{0}^{\left(n\right)}∼2N\left(\epsilon_{F}\right)$ is obtained in the normal state ($T>T_{c}^{⋆}$) from a linear dependence $C\left(T\right)/T=\gamma_{0}+\beta T^{2}$ vs. $T^{2}$ at T=0, $\beta =N\left(12/5\right)\pi^{4}R\theta_{D}^{-3}$, and *N* is the number of atoms in a formula unit. The electron–phonon coupling parameters λ and $\lambda^{⋆}$ characterize the $T_{c}$ and $T_{c}^{⋆}$ phases, respectively. The transition temperature $T_{c}^{⋆}$ ($T_{c}$ for La${}_{3}$Co${}_{4}$Sn${}_{13}$) is defined as *T* at 50% of the normal-state ρ value. In the brackets the $T_{c}^{⋆}$s characterizing the maxima of the f(Δ) function are shown. The final column shows the value of $\frac{dH_{c2}}{dT}$ at $T_{c}$ (in the brackets are the respective data for the inhomogeneous phase $T_{c}^{⋆}$).

Superconductor	Structure	$\gamma_{0}$	$\theta_{D}$	$T_{c}$	$T_{c}^{⋆}$	λ	$\lambda^{⋆}$	$-\frac{{\mathrm{dH}}_{c2}}{\mathrm{dT}}$
		(mJ/mol K${}^{2}$)	(K)	(K)	(K)			$10^{4}\times$ Gs/K
La${}_{3}$Co${}_{4}$Sn${}_{13}$	P$m\bar{3}n$	27.9	220	1.95	-	0.40	-	0.89
La${}_{3}$Rh${}_{4}$Sn${}_{13}$	P$m\bar{3}n$	13.5	173	2.12	2.98 (2.06)	0.43	0.45	0.95 (0.46)
La${}_{3}$Ru${}_{4}$Sn${}_{13}$	P$m\bar{3}n$	9.9	150	1.58	3.76	0.41	0.53	1.29 (0.92)
La${}_{3}$Ru${}_{3}$CoSn${}_{13}$	P$m\bar{3}n$	11.1	166	3.9	6.05 (5.0)	0.52	0.59	1.31 (1.20)
Ca${}_{3}$Rh${}_{4}$Sn${}_{13}$	P$m\bar{3}n$	6.1	213	4.71	4.97	0.51	0.52	0.91 (0.78)
Ca${}_{2.8}$Ce${}_{0.2}$Rh${}_{4}$Sn${}_{13}$	P$m\bar{3}n$	30.0	185	4.53	8.0 (7.4)	0.50	0.59	(0.53)
Ca${}_{2.4}$La${}_{0.6}$Rh${}_{4}$Sn${}_{13}$	P$m\bar{3}n$	38.0	144	4.50	8.1 (7.7)	0.57	0.72	(0.54)
Y${}_{5}$Rh${}_{6}$Sn${}_{18}$	I$4_{1}/acd$	9.3	200	3.08	3.19	0.46	0.47	(1.80)
Y${}_{4.5}$Ca${}_{0.5}$Rh${}_{6}$Sn${}_{18}$	I$4_{1}/acd$	16.3	150	3.10	3.72	0.51	0.52	(1.92)
Lu${}_{5}$Rh${}_{6}$Sn${}_{18}$	I$4_{1}/acd$	18	149	4.06	4.09	0.53	0.54	(1.62)
